# Manufacturing and Characterization of Three-Axis Magnetic Sensors Using the Standard 180 nm CMOS Technology

**DOI:** 10.3390/s21216953

**Published:** 2021-10-20

**Authors:** Chi-Han Wu, Po-Jen Shih, Yao-Chuan Tsai, Ching-Liang Dai

**Affiliations:** 1 Department of Mechanical Engineering, National Chung Hsing University, Taichung 402, Taiwan; abc7755998@smail.nchu.edu.tw; 2 Department of Biomedical Engineering, National Taiwan University, Taipei 106, Taiwan; pjshih@ntu.edu.tw; 3 Department of Bio-Industrial Mechatronics Engineering, National Chung Hsing University, Taichung 402, Taiwan; yctsaii@dragon.nchu.edu.tw

**Keywords:** micro magnetic sensor, three-axis sensing, high sensitivity, CMOS, MEMS

## Abstract

A three-axis micro magnetic sensor (MS) is developed based on the standard 180 nm complementary metal oxide semiconductor (CMOS) technology. The MS designs two magnetic sensing elements (MSEs), which consists of an x/y-MSE and an z-MSE, to reduce cross-sensitivity. The x/y-MSE is constructed by an x-MSE and an y-MSE that are respectively employed to detect in the x- and y-direction magnetic field (MF). The z-MSE is used to sense in the z-direction MF. The x/y-MSE, which is constructed by two magnetotransistors, designs four additional collectors that are employed to increase the sensing current and to enhance the sensitivity of the MS. The Sentaurus TCAD software simulates the characteristic of the MS. The measured results reveal that the MS sensitivity is 534 mV/T in the x-direction MF, 525 mV/T in the y-direction MF and 119 mV/T in the z-axis MF.

## 1. Introduction

Magnetic sensors (MSs) have a wide range of uses and can be applied in control and monitoring devices, industrial products, material testing, manufacturing systems and biomedical engineering. For instance, Lu [1] used a microelectromechanical system (MEMS) MS to develop a location tracking system that was applied in real-time tracking of the vessel location and organ shape for surgical navigation. The location tracking system provided a magnetic field (MF) to a patient and employed the MS node to detect the organ location of the patient. Lee [2] utilized a 3 × 3 array of Hall MSs to construct an MF measurement system for MF mapping. The system could measure the MF distributions of a group of multi-magnets. Oh [3] adopted a small magnet, an MEMS MS and a breathing output component to compose a respiratory monitoring and training system that was used to train and monitor the breath of patients for radiotherapy. Lara-Castro [4] employed a micromachined MS to constitute a portable signal conditioning system for industrial applications to measure ferromagnetic material characteristic. The MS was composed of a silicon resonator, an aluminum loop and four piezoresistors. Krishnapriya [5] manufactured a micro MS with a planar micro coil for biomedical application to detect biomolecules and pathogens. An integrated microfluidic platform, proposed by Feng [6], was assembled by a micro MS and a micro-spiral planar coil. The microfluidic platform was used to detect and manipulate magnetic beads. Zhang [7] designed a magnetic scanning device with a digital micro MS. The magnetic scanning device, which was a non-destructive test, could detect the leakage of MF from steel corrosion in reinforced concrete. An unmanned aerial vehicle (UAV) navigation system, presented by Vetrella [8], was built using an MEMS MS, a global positioning system receiver, an inertial sensor, a navigation algorithm and a vision component. The system was utilized to control and stabilize the UAV flight.

The advantages of micro MS are that is has a small volume, easy integration and a high performance. Recently, the MEMS technology was adopted to design and fabricate various micro MSs. Table 1 summarizes the sensing principle and type for various micro magnetic sensors. For example, an MS, as proposed by Niekiel [9], was fabricated using the MEMS technology. The MEMS MS, which was a magnetic-resonant type, contained a piezoelectric resonator that integrated permanent magnets. Chen [10] developed a MEM MS, which was a giant magneto-impedance type, using surface micromachining. The magnetic sensing material for the sensor was the copper-based amorphous wire. The sensor had a micro coil which surrounded the copper-based amorphous wire, and the output signal for the sensor was produced by the micro coil. The sensitivity of the MEMS MS was 130 mV/Oe. A MEMS MS that was a magnetic-resonant type was presented by Okada [11]. The sensor had a resonator and a micro bridge. The resonator was a silicon bridge with a PZT thin film, and the micro bridge was silicon with a FePd film. When an MF was applied to the micro bridge it produced a deflection to act on the resonator, so that the stiffness of the resonator changed, which lead to a variation in the resonance frequency of the resonator. Guo [12] made an MEMS MS that was a fluxgate type using the micromachining with chemical wet etching. The MEMS MS had a double-layer magnetic core that was deposited by the melt-spinning. Nejad [13] designed an MS. The relation between the mechanical displacement and magnetostatic force for the sensor was analyzed by the governing equation. The MS was made by micromachining, and the MS beams were a triple-layer of gold/Ni/gold. A micromachined MS, developed by Bahreyni [14], was fabricated by bulk micromachining. The sensor, which was a magnetic-resonant type, had an electrostatic resonator. When an MF was supplied to the MS, the resonant frequency of the electrostatic resonator produced a change. The MS did not exhibit hysteresis because it did not use any magnetic materials. Tseng [15] used the CMOS process to fabricate a one-axis micro MS, and the MS sensitivity was 354 mV/T. Sileo [16] employed the microfabrication to make a three-axis Hall MS. The Hall MS structure was constituted by the AlGaAs/InGaAs/GaAs multilayered material. The MS sensitivity was 0.03 V/T. A three-axis MS, presented by Zhao [17], was manufactured through the MEMS technology. The MS had four magnetic transistors and a Hall element. The x-axis, y-axis and z-axis sensitivity for the MS were 77.5 mV/T, 78.6 mV/T and 77.4 mV/T, respectively. Yeh [18] manufactured a three-axis MS, which was a magnetic-piezoelectric type, based on the MEMS technology. The MS structure had a silicon diaphragm and a magnetic nickel thick film and a piezoelectric lead zirconate titanate (PZT) thin film located on the silicon diaphragm. When the magnetic nickel film was excited by an alternating current MF, the PZT film and the silicon diaphragm generated a vibration and displacement. The PZT film produced an output voltage. The x-axis, y-axis and z-axis sensitivity for the MS were 0.156 mV/Oe, 0.156 mV/Oe, and 0.035 mV/Oe, respectively. These sensors [9,10,11,12,13,14,15] were one-axis MSs. The sensors [16,17,18] were three-axis MSs. This work develops a three-axis micro MS, in which its sensitivity exceeds that of the sensors [16,17]

The CMOS technology is adopted to make micro elements [19,20], micro actuators [21,22,23,24] and micro sensors [25,26,27,28,29,30]. The benefits of MSs that are made using the CMOS technology have low noise, high performance and easy mass-production. We employ this technology to design and manufacture a three-axis micro MS. To enhance the sensitivity and decrease the cross-sensitivity, the MS is built by two magnetic sensing elements (MSEs) that are an x/y-MSE and a z-MSE. The x/y-MSE senses in the x- and y-direction MF, and the z-MSE detects in the z-direction MF.

## 2. Design of Magnetic Sensor

The three-axis micro MS includes an x/y-MSE and an z-MSE. The x/y-MSE is combined by an x-MSE and a y-MSE. The x-MSE and y-MSE are used to detect the MF in the x-direction and the y-direction, respectively. The z-MSE is employed to measure in the z-direction MF. 

**Figure 1:**
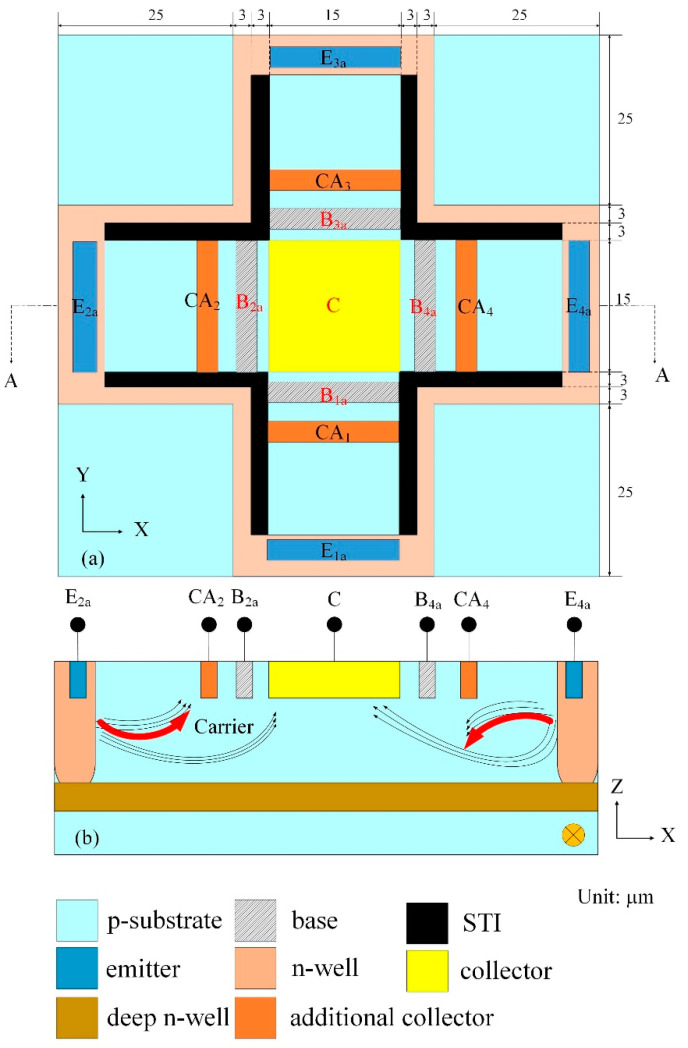
(**a**) x/y magnetic sensing element (MSE) structure; (**b**) Cross-sectional view for x/y-MSE along line AA.

Figure 1a displays the x/y-MSE structure, where E_1a_, E_2a_, E_3a_, and E_4a_ are emitters; B_1a_, B_2a_, B_3a_ and B_4a_ are bases; CA_1_, CA_2_, CA_3_ and CA_4_ are additional collectors; C is a collector; STI is shallow trench isolation. The STI is utilized to restrict the current moving direction and to decrease the current leakage. The x-MSE that is a magnetic transistor is constructed by the emitters E_1a_ and E_3a_, the additional collectors CA_1_ and CA_3_, the bases B_1a_ and B_3a_, and the collector C. The y-MSE is a symmetric structure with the x-MSE, and the y-MSE is composed of the emitters E_2a_ and E_4a_, the additional collectors CA_2_ and CA_4_, the bases B_2a_ and B_4a_, and the collector C.

The y-MSE cross-sectional view is illustrated in Figure 1b. When the bias applies to the collector C, additional collectors (CA_2_ and CA_4_) and bases (B_2a_ and B_4a_) and carriers move from the emitters (E_2a_ and E_4a_) to the collector C, bases (B_2a_ and B_4a_) and additional collectors (CA_2_ and CA_4_). There is an MF in the y-direction. The current and MF generate a Lorentz force that acts on carriers, resulting in carriers (on the right in Figure 1b) are bended downward. Most carriers migrate to the collector C, such that the current of the additional collector CA_4_ decreases. Opposite, carriers (on the left in Figure 1b) are bended upward by the Lorentz force. Most carriers migrate to the additional collector CA_2_, leading to an increase in the current of the additional collector CA_2_. Therefore, when the y-direction MF applies to the x/y-MSE, the x/y-MSE generates a voltage difference between the additional collectors CA_2_ and CA_4_. The output voltage (Vo) of the MS is obtained by the voltage difference of the additional collectors CA_2_ and CA_4_ when applying a y-direction MF to the x/y-MSE.

As shown in Figure 1b, the element structure of the x-MSE is similar to that of the y-MSE. The structure of the x-MSE, which consists of the emitters (E_1a_ and E_3a_), the bases (B_1a_ and B_3a_), the additional collectors (CA_1_ and CA_3_) and the collector C, is along the y-axis direction. When the bias applies to the collector C, additional collectors (CA_1_ and CA_3_) and bases (B_1a_ and B_3a_) and carriers move from the emitters (E_1a_ and E_3a_) to the collector C, bases (B_1a_ and B_3a_) and additional collectors (CA_1_ and CA_3_). Suppose that there is an MF in the x-direction, then the current and the MF generate a Lorentz force that acts on carriers, such that carriers, which move from the emitter E_1a_ to the base B_1a_ and collector C, are bended downward. Most carriers migrate to the collector C, leading to a decrease in the current of the additional collector CA_1_. Opposite, carriers that move from the emitter E_3a_ to the collector C and base B_3a_ are deflected upward by the Lorentz force. Most carriers migrate to the additional collector CA_3_, such that the current of the additional collector CA_2_ increases. Therefore, when the x-direction, MF applies to the x/y-MSE, the x/y-MSE produces a voltage difference between the additional collectors CA_1_ and CA_3_. The MS Vo is obtained by the voltage difference of the additional collectors CA_1_ and CA_3_ when applying the x-direction MF to the x/y-MSE.

**Figure 2:**
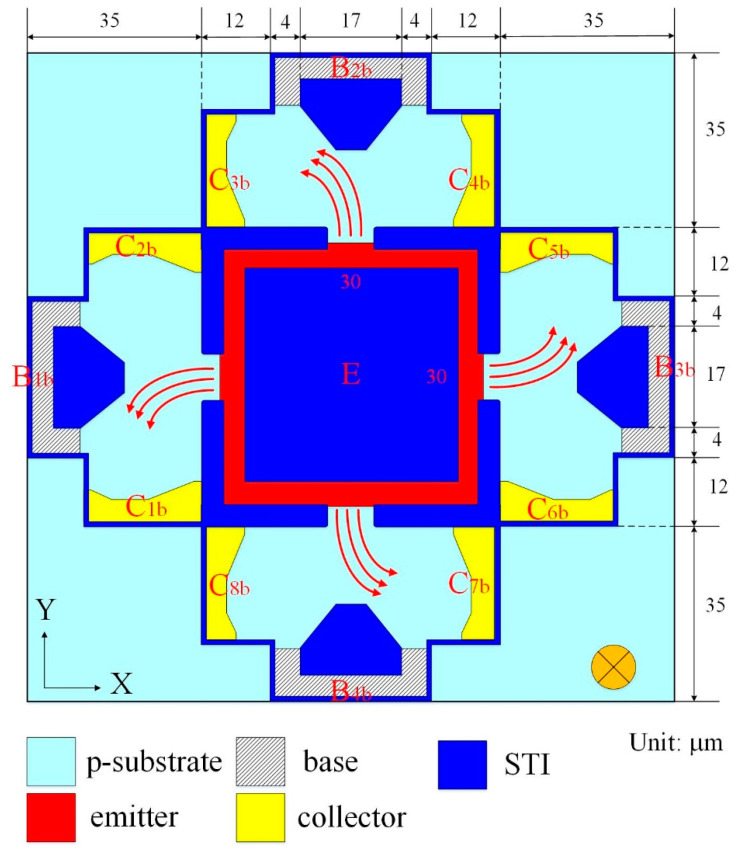
The z-MSE structure, where E is emitter; B_1b_, B_2b_, B_3b_ and B_4b_ are base; C_1b_, C_2b_, C_3b_, C_4b_, C_5b_, C_6b_, C_7b_ and C_8b_ are collectors.

Figure 2 displays the z-MSE structure of the MS. The z-MSE is composed of one emitter E, four bases and eight collectors. The STI oxide is used to restrict the current moving direction and reduces the current leakage. When the bias applies to the bases and the collectors, carriers migrate from the emitter to the bases and collectors. There is an MF in the z-direction. The current and the MF produces a Lorentz force that acts on carriers, such that carriers are bended toward the collectors (C_1b_, C_3b_, C_5b_ and C_7b_), resulting in the currents of the collectors (C_1b_, C_3b_, C_5b_ and C_7b_) being higher than that of the collectors (C_2b_, C_4b_, C_6b_ and C_8b_). A voltage difference is generated between the collectors. The Vo of the MS is obtained by the voltage differences in series when applying a z-direction MF to the z-MSE.

**Figure 3:**
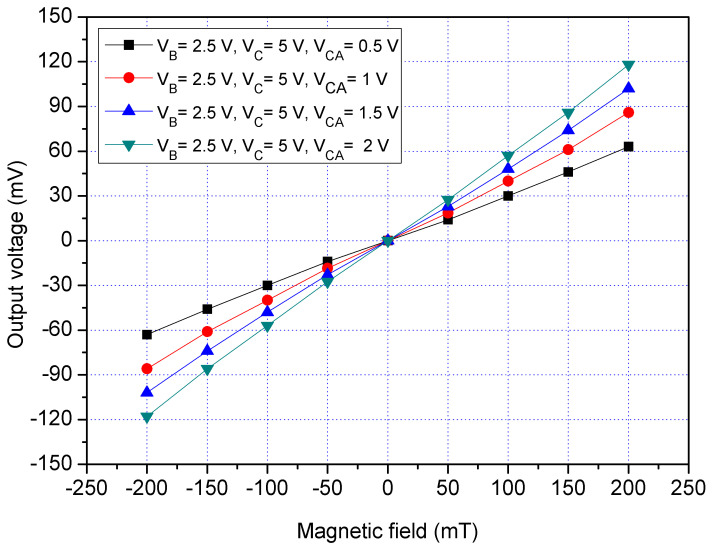
Simulated output voltage (Vo) in the x-direction MF.

The performance of the x/y-MSE was simulated using the Sentaurus TCAD software. The x/y-MSE model (Figure 1a) was set, and the Delaunay triangulation method was used to mesh the x/y-MSE model. The Poisson electron hole approach was employed to evaluate the electrical and MF coupling effect for the x/y-MSE. The Bank–Rose approach was employed to compute the distribution of carrier density for the x/y-MSE. Figure 3 presents the simulated Vo for the MS in the x-direction MF, where V_B_ is the bias of the bases; V_C_ is the bias of the collector and V_CA_ is the bias of the additional collectors. In the evaluation, the collector C and additional collectors (CA_1_, CA_2_, CA_3_ and CA_4_), respectively, connected with a resistance of 1 kΩ. The bases bias was 2.5 V, and the collector bias was 5 V. The bias of the additional collectors was with different voltages of 0.5, 1, 1.5 and 2 V. The x/y-MSE was applied by the x-direction MF, and the x/y-MSE Vo was the voltage difference of the additional collector AC_1_/AC_3_. The evaluated results depicted that the MS Vo changed from −116 mV at −200 mT to 116 mV at 200 mT when V_B_ = 2.5 V, V_C_ = 5 V and V_CA_ =2 V. The slope of the curve (at V_B_ = 2.5 V, V_C_ = 5 V and V_CA_ =2 V) was 580 mV/T, so the evaluated sensitivity of the x/y-MSE was 580 mV/T at V_B_ = 2.5 V, V_C_ = 5 V and V_CA_ = 2 V in the x-direction MF. 

**Figure 4:**
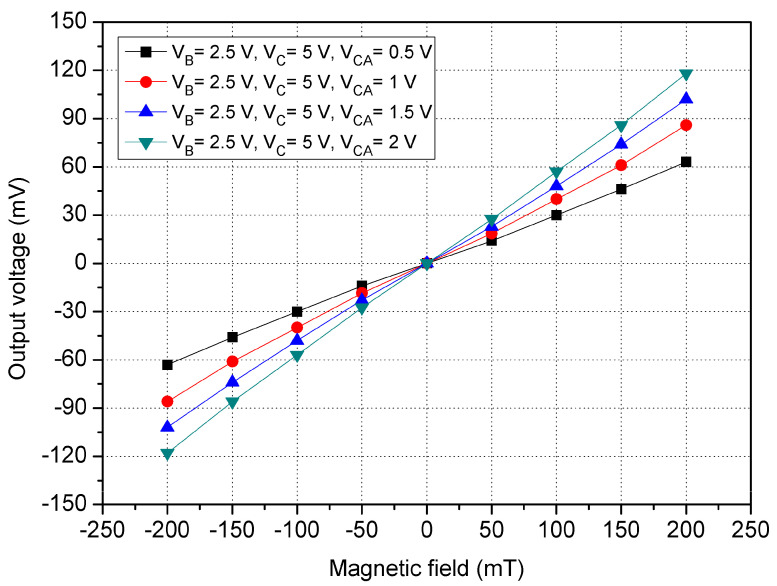
Simulated Vo in the y-direction MF.

The x/y-MSE was applied by the y-direction MF, and the Vo of the x/y-MSE was the voltage difference of the additional collector AC_2_/AC_4_. Figure 4 shows the x/y-MSE Vo in the y-direction MF, where V_B_ is the bias of the bases; V_C_ is the bias of the collector and V_CA_ is bias of the additional collectors. The results presented that the MS Vo increased from −116 mV at −200 mT to 116 mV at 200 mT when V_B_ = 2.5 V, V_C_ = 5 V and V_CA_ = 2 V. The slope of the curve (at V_B_ = 2.5 V, V_C_ = 5 V and V_CA_ =2 V) was 580 mV/T, so the evaluated sensitivity for the x/y-MSE was 580 mV/T at V_B_ = 2.5 V, V_C_ = 5 V and V_CA_ = 2 V in the y-direction MF. The evaluated results of the x/y-MSE in the y-direction MF was the same with that in the x-direction MF because the x/y-MSE structure was a symmetric. 

**Figure 5:**
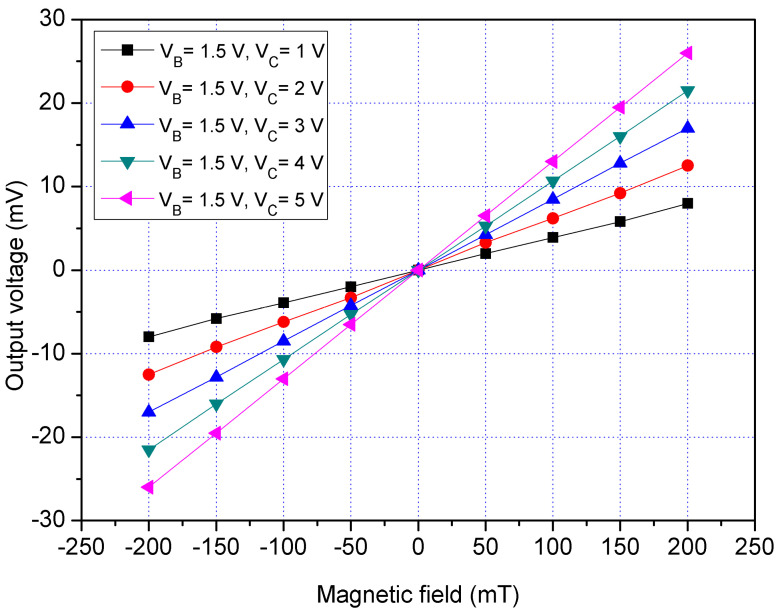
Simulated Vo in the z-direction MF.

The performance of the z-MSE was simulated using the Sentaurus TCAD. The z-MSE model was established according to the structure in Figure 2, and the z-MSE Vo was evaluated using the same simulation approach. In the simulation, the bases and collectors connected with a resistance of 1 kΩ, respectively. The bias of the bases was 1.5 V, the bias of the collectors was 5 V. The z-MSE was supplied by the z-direction MF, and the z-MSE Vo was computed by the Sentaurus TCAD. Figure 5 shows the z-MSE Vo in the z-direction MF, where V_B_ is bias of the bases and V_C_ is bias of the collector. The evaluated results depicted that the z-MSE Vo changed from −26 mV at −200 mT to 26 mV at 200 mT when V_B_ = 1.5 V and V_C_ = 5 V. The slope of the curve at V_B_ = 2.5 V and V_C_ = 5 V was 130 mV/T, so the evaluated sensitivity of the z-MSE was 130 mV/T at V_B_ = 1.5 V and V_C_ = 5 V.

**Figure 6:**
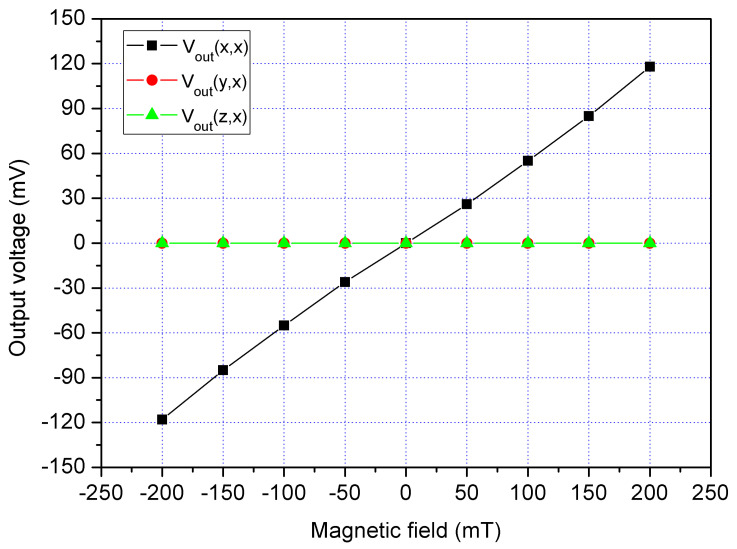
Simulation of output for the MS in the x-direction MF.

**Figure 7:**
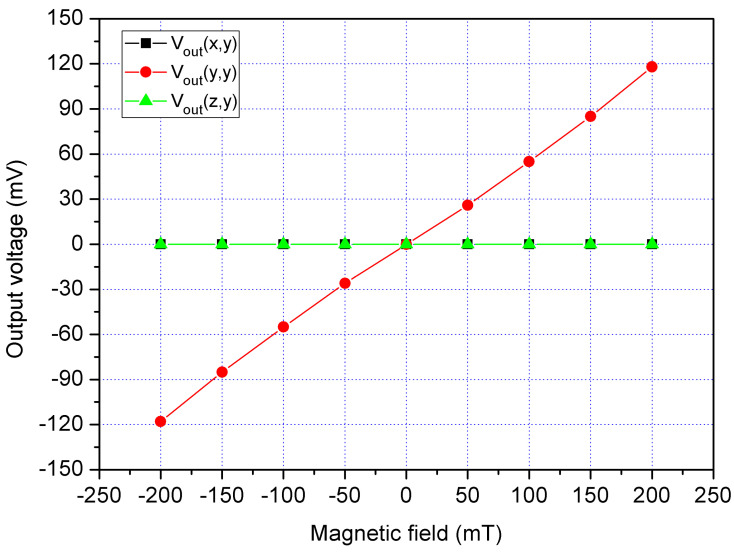
Simulation of output for the MS in the y-direction MF.

**Figure 8:**
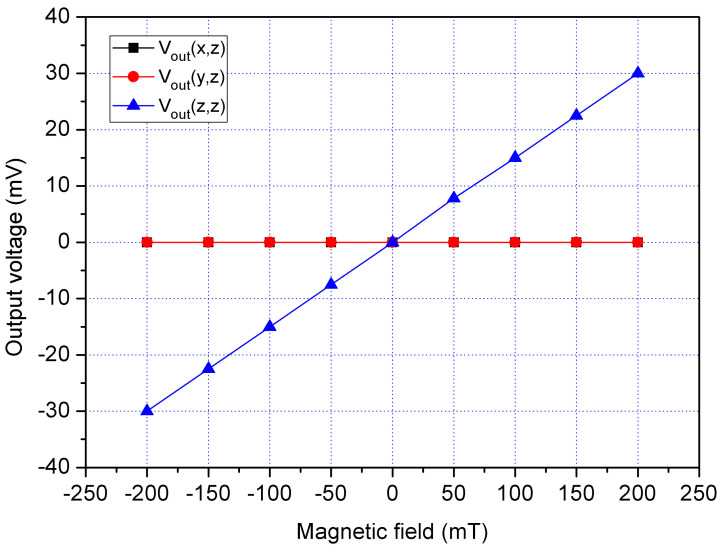
Simulation of output for the MS in the z-direction MF.

In order to characterize the MS cross-sensitivity, the cross-sensitivity of x/y-MSE and z-MSE were simulated using the Sentaurus TCAD with the same simulation approach. The bias of bases for the x/y-MSE was 2.5 V, and the bias of additional collectors for the x/y-MSE was 2 V. The bias of collector for the x/y-MSE was 5 V. At the same time, the bias of bases for the z-MSE was 1.5 V, and the bias of collectors for the z-MSE was 5 V. First, the Vo of the x/y-MSE and z-MSE was computed when applying an x-direction MF to the MS. Figure 6 shows the evaluated output for the MS, where V_out_(x,x) is the x-axis Vo for x/y-MSE in the x-direction MF; V_out_(y,x) is the y-axis Vo for x/y-MSE Vo in the x-direction MF; V_out_(z,x) is the z-MSE Vo in the x-direction MF. The results presented that the V_out_(y,x) and V_out_(z,x) values approximated to zero and the V_out_(x,x) had a high response, so the MS cross-sensitivity in the x-direction MF was very small. Then, the Vo of the x/y-MSE and z-MSE was calculated when applying a y-direction MF to the MS. Figure 7 shows the evaluated output for the MS, where V_out_(x,y) is the x-axis Vo for x/y-MSE in the y-direction MF; V_out_(y,y) is the y-axis Vo for x/y-MSE in the y-direction MF; V_out_(z,y) is the z-MSE Vo in the y-direction MF. The results revealed that the V_out_(x,y) and V_out_(z,y) values approximated zero, and the V_out_(y,y) had a high response. The MS cross-sensitivity in the y-direction MF was very small. Finally, the Vo of the x/y-MSE and z-MSE was simulated when applying a z-direction MF to the MS. Figure 8 shows the evaluated output for the MS, where V_out_(x,z) is the x-axis Vo for x/y-MSE in the z-direction MF; V_out_(y,z) is the y-axis Vo for x/y-MSE in the z-direction MF; V_out_(y,z) is the z-MSE Vo in the z-direction MF. The results depicted that the V_out_(y,y) had a high response, and the V_out_(x,y) and V_out_(z,y) values approximated zero. The MS cross-sensitivity in the z-direction MF was very small. 

## 3. Manufacturing of Magnetic Sensor 

The three-axis MS contained an x/y-MSE and a z-MSE. The x/y-MSE (Figure 1) was composed of four emitters, four bases, four additional collectors and one collector, where the emitters, collector, additional collectors were n-type silicon with doping phosphorus and the bases were p-type silicon with doping boron. The deep n-well layer was used to restrict the current movement range and to decrease current leakage. 

**Figure 9:**
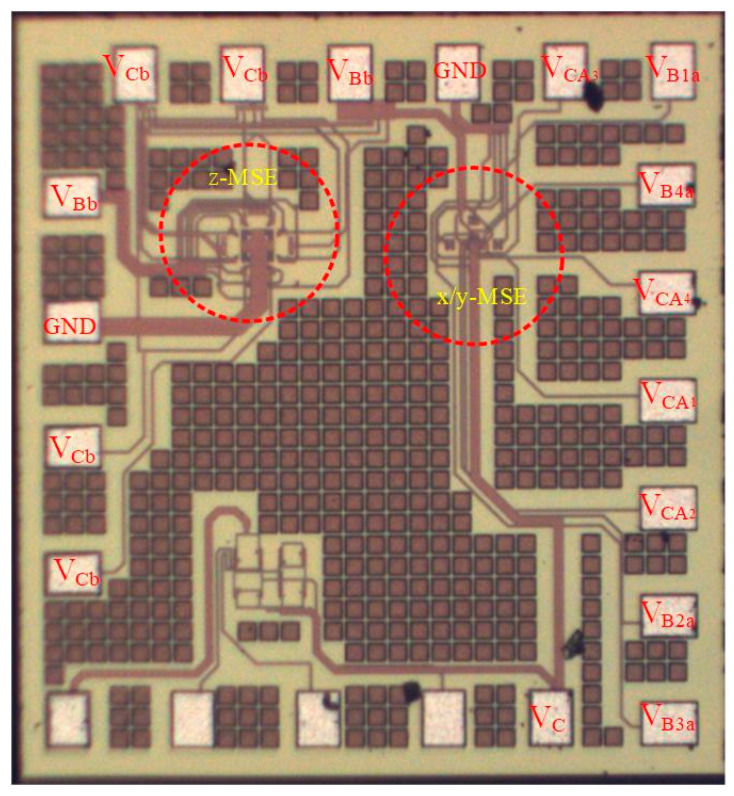
Picture of the MS chip.

**Figure 10:**
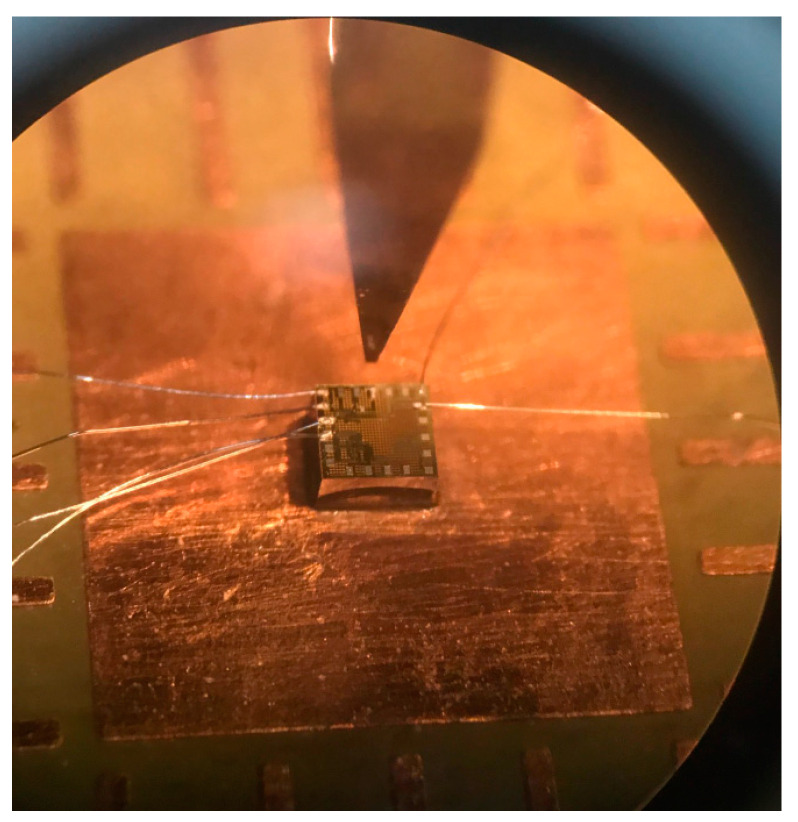
Picture of the MS chip in wire-bonding.

As shown in Figure 2, the z-MSE was composed of four bases, eight collectors and one emitter, where the bases were p-type silicon with doping boron and the collectors and emitters were n-type silicon with doping phosphorus. According to the x/y-MSE structure (Figure 1) and the z-MSE structure (Figure 2), the x/y-MSE and z-MSE layouts were devised. In accordance with the x/y-MSE and z-MSE layout, the three-axis MS was fabricated based on the standard CMOS process from Taiwan Semiconductor Manufacturing Company (TSMC) [31]. Figure 9 shows a picture of the three-axis MS after the CMOS process. The three-axis MS chip (Figure 9) included an z-MSE and an x/y-MSE. Figure 10 demonstrates a picture of the three-axis MS chip during the wire-bonding. The three-axis MS chip was bonded on a printed circuit board by a wire-bonder for measuring its characteristics.

## 4. Results

**Figure 11:**
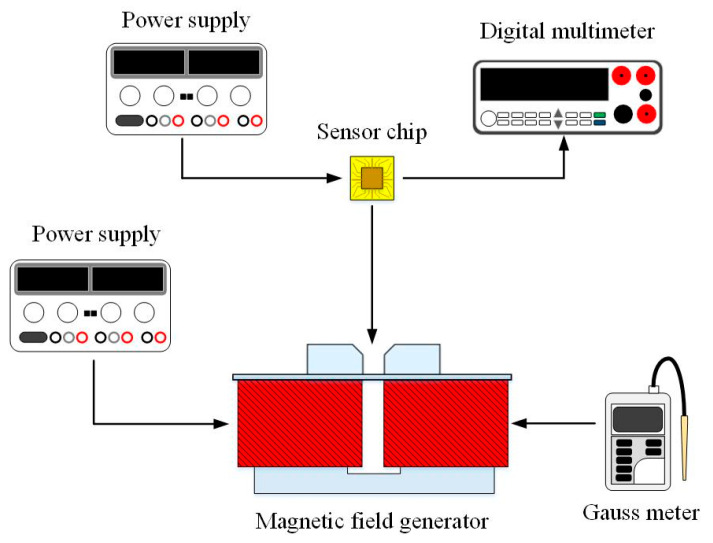
Measurement setup for the MS.

The three-axis MS was measured utilizing a digital multimeter, two power supplies, a Gauss meter and an MF generator. Figure 11 demonstrates the setup for measuring the three-axis MS characteristic. The MS chip was placed in the MF generator. The power supply inputted power to the MF generator. The Gauss meter calibrated the MF magnitude that was produced by the MF generator. The MF generator supplied various MFs for the MS measurement. The power supply provided the bias for the MS. The digital multimeter measured the Vo of the three-axis MS. 

**Figure 12:**
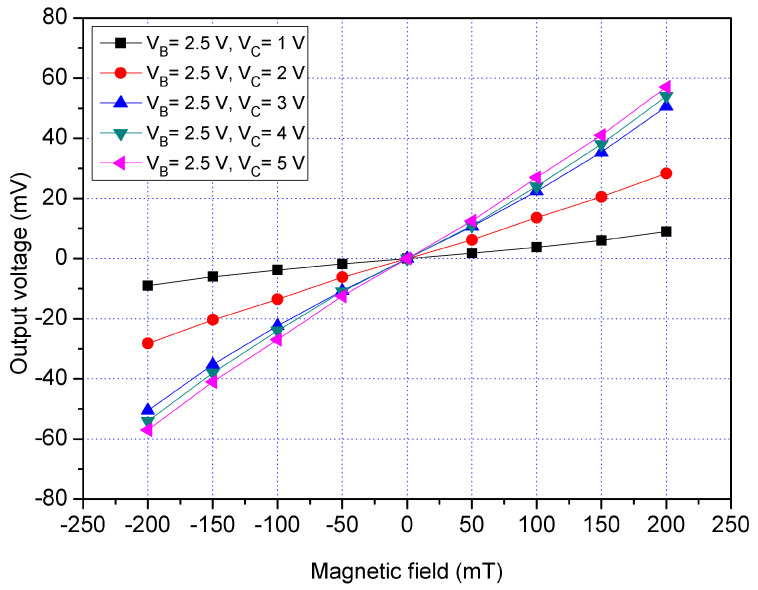
Measured Vo for the x/y-MSE without bias of the additional collectors in the x-direction MF.

The x/y-MSE characteristic was tested in the x-direction MF. The MS chip (Figure 11) was placed in the MF generator. An MF in the x-direction that was provided by the MF generator was applied to the x/y-MSE. A bias of 2.5 V was supplied to the bases of x/y-MSE. The additional collectors were without bias. The collector and each additional collector, respectively, was connected with a resistance of 1 kΩ. The different voltages including 1, 2, 3, 4 and 5 V were supplied to the collector of x/y-MSE. The x/y-MSE Vo that was the voltage difference between the addition collectors CA_1_ and CA_3_ was recorded by the digital multimeter. The tested results for the x/y-MSE without the additional collectors bias in the x-direction MF are shown in Figure 12, where V_B_ is the bias of the bases and V_C_ is the bias of the collector. When V_B_ = 2.5 V and V_C_ = 1 V, the x/y-MSE Vo varied from −9.1 mV at −200 mT to 9.2 mV at 200 mT. When V_B_ = 2.5 V and V_C_ = 3 V, the x/y-MSE Vo increased from −50.5 mV at −200 mT to 50.6 mV at 200 mT. When V_B_ = 2.5 V and V_C_ = 5 V, the x/y-MSE Vo changed from −57.2 mV at −200 mT to 57.1 mV at 200 mT. The curve slope was 286 mV/T at V_B_ = 2.5 V and V_C_ = 5 V, so the x/y-MSE sensitivity was 286 mV/T at V_B_ = 2.5 V and V_C_ = 5 V in the x-direction MF. 

**Figure 13:**
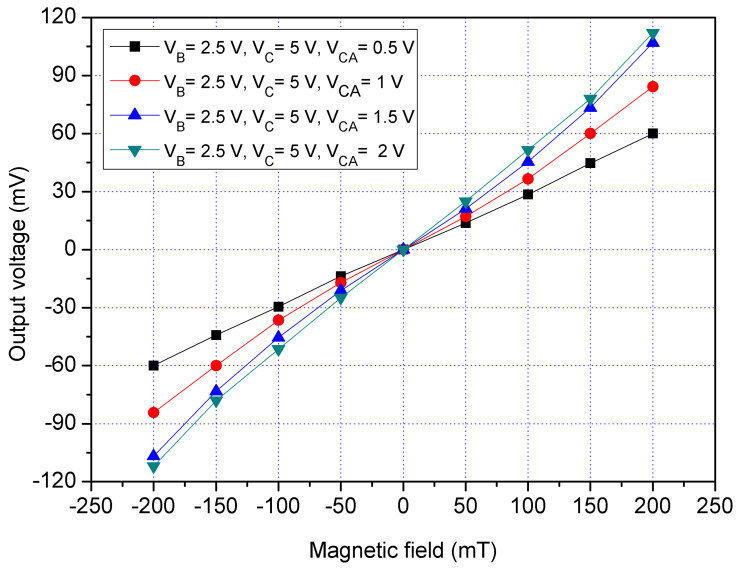
Measured Vo for the x/y-MSE with bias of the additional collectors in the x-direction MF.

The x/y-MSE had the addition collectors that were used to increase the moving current and enhance the x/y-MSE sensitivity. To characterize the function of additional collectors, the x/y-MSE was measured under different biases of additional collectors. A bias of 2.5 V was provided to the bases of the x/y-MSE, and a bias of 5 V was applied to the collector of the x/y-MSE. The difference voltages that included 0.5, 1, 1.5 and 2 V were applied to the additional collectors of the x/y-MSE. The Vo of the x/y-MSE was recorded by a digital multimeter. Figure 13 displays the measured Vo for the x/y-MSE with the additional collectors bias in the x-direction MF, where V_B_ is bias of the bases; V_C_ is bias of the collector and V_CA_ is bias of the additional collectors. When V_B_ = 2.5 V, V_C_ = 5 V and V_CA_ = 0.5 V, the x/y-MSE Vo varied from −60.3 mV at −200 mT to 60.2 mV at 200 mT. When V_B_ = 2.5 V, V_C_ = 5 V and V_CA_ = 1 V, the x/y-MSE Vo increased from −84.3 mV at −200 mT to 84.4 mV at 200 mT. When V_B_ = 2.5 V, V_C_ = 5 V and V_CA_ = 2 V, the x/y-MSE Vo changed from −112 mV at −200 mT to 112 mV at 200 mT. The linear regression method was used to fit the curve at V_B_ = 2.5 V, V_C_ = 5 V and V_CA_ = 2 V. The results showed that the regression line had a slope of 534 mV/T and a coefficient of determination R^2^ = 0.9984, so the sensitivity of the x/y-MSE was 534 mV/T at V_B_ = 2.5 V, V_C_ = 5 V and V_CA_ = 2 V in the x-direction MF. The output linearity for the x-MSE was 99%. In a comparison of results in Figure 12 and Figure 13, the Vo of the x/y-MSE with the additional collectors bias exceeds that of the x/y-MSE without the additional collectors bias. In the x-direction MF, the sensitivity of the x/y-MSE (534 mV/T at V_B_ = 2.5 V, V_C_ = 5 V and V_CA_=2 V) with the addition of collectors bias is higher than that of the x/y-MSE (286 mV/T at V_B_ = 2.5 V and V_C_ = 5 V) without the additional collectors bias. 

**Figure 14:**
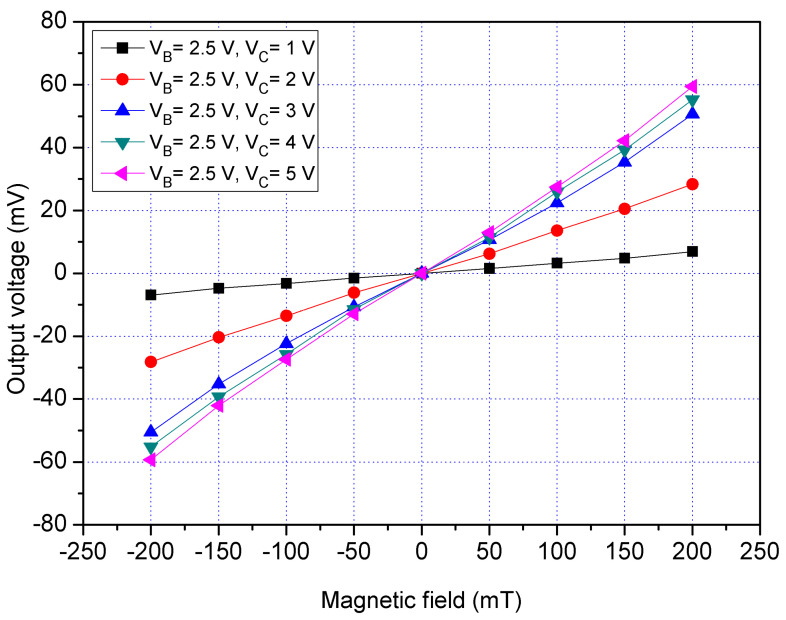
Measured Vo for the x/y-MSE without bias of the additional collectors in the y-direction MF.

The x/y-MSE characteristic was tested in the y-direction MF. As shown in Figure 11, the MS chip was placed in the MF generator. An MF in the y-direction that was produced by the MF generator was supplied to the x/y-MSE. The collector and each additional collector connected with a resistance of 1 kΩ, respectively. A bias of 2.5 V was applied to the bases of the x/y-MSE. The additional collectors were without bias. The difference in voltages that had 1, 2, 3, 4 and 5 V were provided to the collector of the x/y-MSE. The Vo of the x/y-MSE that was the voltage difference between the addition collectors CA_2_ and CA_4_ and were recorded by the digital multimeter. The tested results for the x/y-MSE without the additional collectors bias in the y-direction MF is shown in Figure 14, where V_B_ is the bias of the bases and V_C_ is the bias of the collector. When V_B_ = 2.5 V and V_C_ = 1 V, the x/y-MSE Vo varied from −8.9 mV at −200 mT to 9 mV at 200 mT. When V_B_ = 2.5 V and V_C_ = 3 V, the x/y-MSE Vo increased from −50.9 mV at −200 mT to 50.8 mV at 200 mT. When V_B_ = 2.5 V and V_C_ = 5 V, the x/y-MSE Vo changed from −56.9 mV at −200 mT to 56.8 mV at 200 mT. The slope of curve was 283 mV/T at V_B_ = 2.5 V and V_C_ = 5 V, so the sensitivity of the x/y-MSE was 283 mV/T at V_B_ = 2.5 V and V_C_ = 5 V in the y-direction MF. 

**Figure 15:**
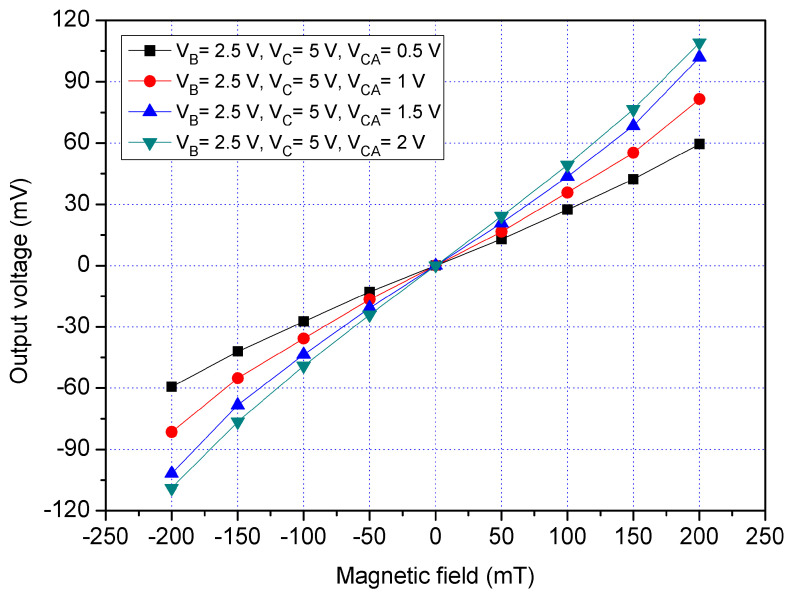
Measured Vo for the x/y-MSE with bias of the additional collectors in the y-direction MF.

To understand the function of the additional collectors, the x/y-MSE was tested under the different biases of additional collectors. A bias of 2.5 V was applied to the bases of the x/y-MSE, and a bias of 5 V was supplied to the collector of the x/y-MSE. The different voltages that had 0.5, 1, 1.5 and 2 V were provided to the additional collectors of the x/y-MSE. The Vo of the x/y-MSE was measured by the digital multimeter. Figure 15 displays the measured Vo for the x/y-MSE with the additional collectors bias in the y-direction MF, where V_B_ is the bias of the bases; V_C_ is the bias of the collector and V_CA_ is the bias of the additional collectors. When V_B_ = 2.5 V, V_C_ = 5 V and V_CA_ = 0.5 V, the x/y-MSE Vo varied from −59.5 mV at −200 mT to 59.6 mV at 200 mT. When V_B_ = 2.5 V, V_C_ = 5 V and V_CA_ = 1 V, the x/y-MSE Vo increased from −83.6 mV at −200 mT to 83.5 mV at 200 mT. When V_B_ = 2.5 V, V_C_ = 5 V and V_CA_ = 2 V, the x/y-MSE Vo changed from −111.5 mV at −200 mT to 111.5 mV at 200 mT. The linear regression method was employed to fit the curve at V_B_ = 2.5 V, V_C_ = 5 V and V_CA_ = 2 V. The results depicted that the regression line had a slope of 525 mV/T and a coefficient of determination R^2^ = 0.9982, so the sensitivity of the x/y-MSE was 525 mV/T at V_B_ = 2.5 V, V_C_ = 5 V and V_CA_ = 2 V in the y-direction MF. The output linearity for the y-MSE was 99%. A comparison of results in Figure 14 and Figure 15, the Vo of the x/y-MSE with the additional collectors bias exceeds that of the x/y-MSE without the additional collectors bias. In the y-direction MF, the sensitivity of the x/y-MSE (525 mV/T at V_B_ = 2.5 V, V_C_ = 5 V and V_CA_ = 2 V) with the addition collector bias exceeds that of the x/y-MSE (283 mV/T at V_B_ = 2.5 V and V_C_ =5 V) without the additional collectors bias.

**Figure 16:**
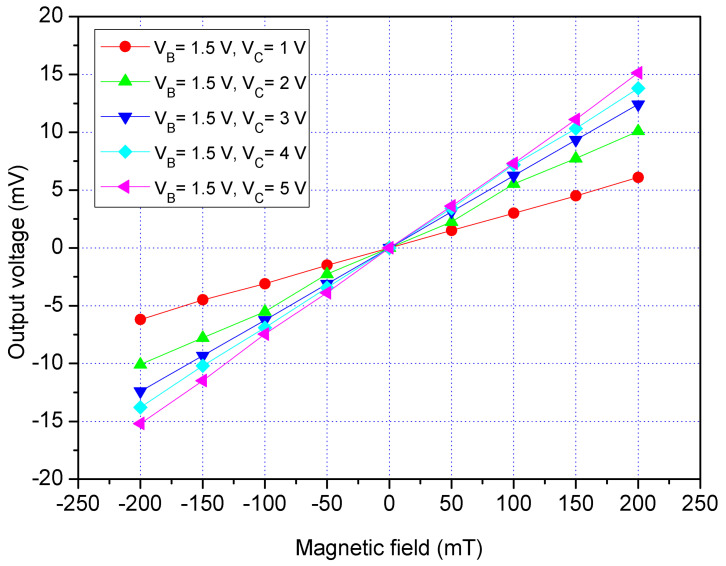
Measured Vo for the z-MSE at V_B_ = 1.2 V in the z-direction MF.

**Figure 17:**
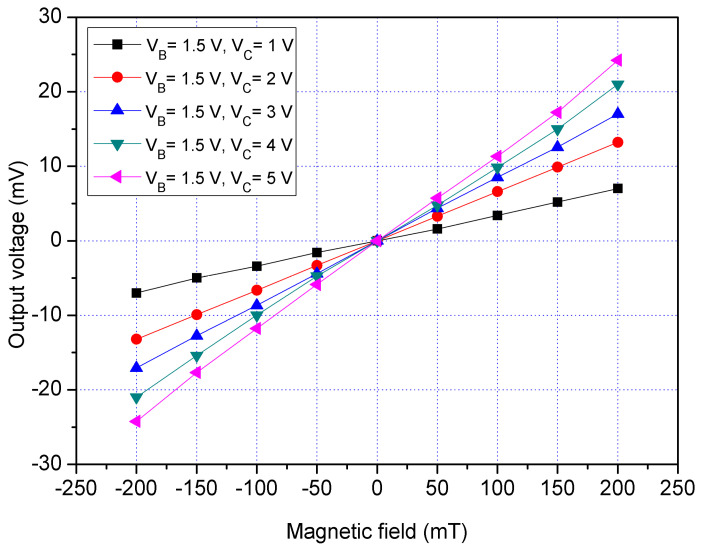
Measured Vo for the z-MSE at V_B_ = 1.5 V in the z-direction MF.

The z-MSE characteristic was tested in the z-direction MF. The MF generator produced a z-direction MF that applied to the z-MSE. The power supply provided a bias of 1.2 V to the bases of the z-MSE. The difference voltages that had 1, 2, 3, 4 and 5 V were applied to the collectors of the z-MSE. The bases and collectors were connected with a resistance of 1 kΩ, respectively. The Vo of the z-MSE was detected by the digital multimeter. Figure 16 displays the measured Vo for the z-MSE at V_B_ = 1.2 V in the z-direction MF, where V_B_ is bias of the bases and V_C_ is bias of the collectors. When V_B_ = 1.2 V and V_C_ = 1 V, the z-MSE Vo increased from −6.2 mV at −200 mT to 6.1 mV at 200 mT. When V_B_ = 1.2 V and V_C_ = 3 V, the z-MSE Vo changed from −12.4 mV at −200 mT to 12.4 mV at 200 mT. When V_B_ = 1.2 V and V_C_ = 5 V, the z-MSE Vo varied from −15.2 mV at −200 mT to 15.1 mV at 200 mT. The slope of curve was 75 mV/T at V_B_ = 1.2 V and V_C_ = 5 V, so the sensitivity of the x/y-MSE was 75 mV/mT at V_B_ = 1.2 V and V_C_ = 5 V in the z-direction MF. To characterize the influence of the bases voltage for the z-MSE, the bias of the bases was increased to 1.5 V. The difference voltages that included 1, 2, 3, 4 and 5 V were supplied to the collectors of the z-MSE. Figure 17 shows the measured Vo for the z-MSE at V_B_ = 1.5 V in the z-direction MF, where V_B_ is the bias of the bases and V_C_ is the bias of the collectors. When V_B_ = 1.5 V and V_C_ = 1 V, the z-MSE Vo changed from −7.5 mV at −200 mT to 7.6 mT at 200 mV. When V_B_ = 1.5 V and V_C_ = 3 V, the z-MSE Vo varied from −17.3 mV at −200 mT to 17.3 mT at 200 mV. When V_B_ = 1.5 V and V_C_ = 5 V, the z-MSE Vo increased from −24.2 mV at −200 mT to 24.1 mV at 200 mT. The linear regression method was used to fit the curve at V_B_ = 1.5 V and V_C_ = 5 V. The results showed that the regression line had a slope of 119 mV/T and a coefficient of determination R^2^ = 0.9995, so the sensitivity of the z-MSE was 119 mV/T at V_B_ = 1.5 V and V_C_ = 5 V in the z-direction MF. The output linearity for the z-MSE was 99%. A comparison of results in Figure 16 and Figure 17, the Vo of the z-MSE at V_B_ = 1.5 V exceeds that of the z-MSE at V_B_ = 1.2 V. In the z-direction MF, and the sensitivity of the z-MSE increases from 75 mV/T at V_B_ = 1.2 V and V_C_ = 5 V to 119 mV/T at V_B_ = 1.5 V and V_C_ = 5 V. 

**Figure 18:**
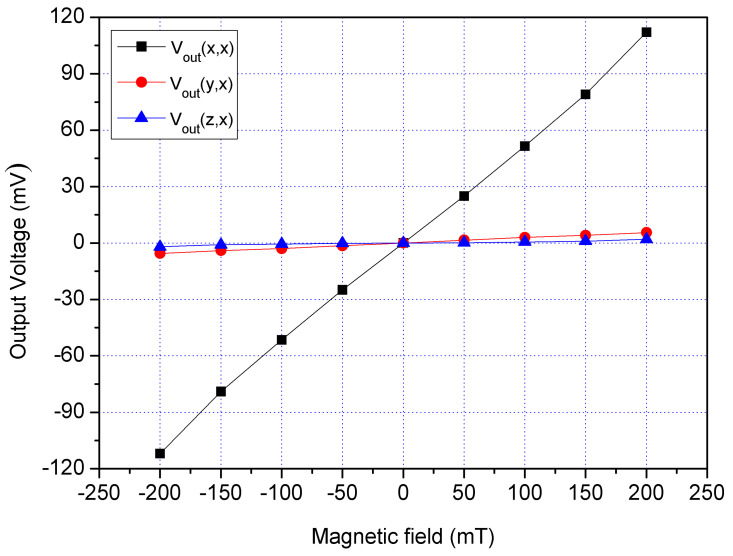
Measured Vo for the MS in the x-direction MF.

An excellent three-axis MS must have a low cross-sensitivity. The cross-sensitivity of the MS was investigated. First, an x-direction MF was applied to the MS. A bias of 2.5 V was supplied to the bases of x/y-MSE. A bias of 5 V was provided to the collector of x/y-MSE, and a bias of 2 V was applied to the additional collectors of x/y-MSE. At the same time, a bias of 1.5 was supplied to the bases of z-MSE, and a bias of 5 V was provided to the collectors of z-MSE. The digital multimeter measured the Vo of the x/y-MSE and z-MSE. Figure 18 displays three-axis Vo for the MS in the x-direction magnetic, where V_out_(x,x) is the x-axis Vo for the x/y-MSE in the x-direction MF; V_out_(y,x) is the y-axis Vo for the x/y-MSE in the x-direction MF and V_out_(z,x) is the z-MSE Vo in the x-direction MF. The V_out_(y,x) and V_out_(z,x) values in Figure 18 are very small. The slope of the curve V_out_(y,x) was 25.4 mV/T, and the slope of the curve V_out_(z,x) was 11.2 mV/T. In the x-direction MF, the MS had a cross-sensitivity of 25.4 mV/T (y-axis output) and a cross-sensitivity of 11.2 mV/T (z-axis output). The MS sensitivity in the x-direction MF was 534 mV/T, so the MS cross-sensitivity in x-direction MF was less than 4.8%.

**Figure 19:**
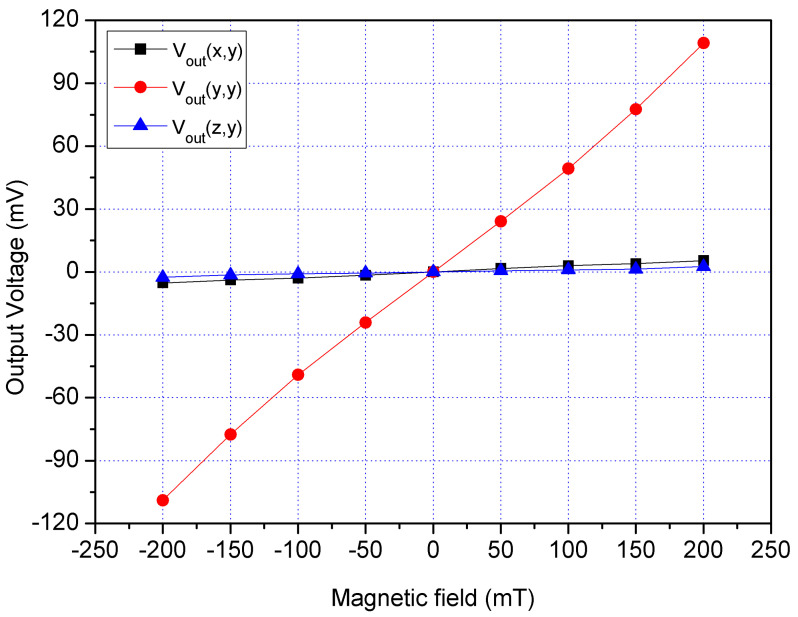
Measured Vo for the MS in the y-direction MF.

A y-direction MF was provided to the MS. A bias of 2.5 V was provided to the bases of x/y-MSE. A bias of 5 V was supplied to the collector of x/y-MSE, and a bias of 2 V was applied to the additional collectors of x/y-MSE. At the same time, a bias of 1.5 was applied to the bases of z-MSE, and a bias of 5 V was supplied to the collectors of z-MSE. The digital multimeter recorded the Vo of the x/y-MSE and z-MSE. Figure 19 displays three-axis Vo for the MS in the y-direction magnetic, where V_out_(x,y) is the x-axis Vo for the x/y-MSE in the y-direction MF; V_out_(y,y) is the y-axis Vo for the x/y-MSE in the y-direction MF and V_out_(z,y) is the z-MSE Vo in the y-direction MF. The V_out_(x,y) and V_out_(z,y) values in Figure 19 are very low. The slope of the curve V_out_(x,y) was 24.5 mV/T, and the slope of the curve V_out_(z,y) was 12 mV/T. In the y-direction MF, the MS had a cross-sensitivity of 24.5 mV/T (x-axis output) and a cross-sensitivity of 12 mV/T (z-axis output). The MS sensitivity in the y-direction MF was 525 mV/T, so the MS cross-sensitivity in y-direction MF was less than 4.7%.

**Figure 20:**
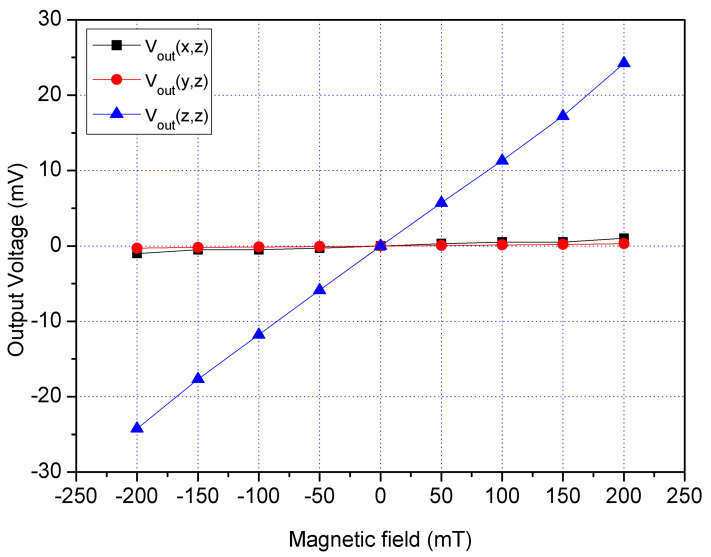
Measured Vo for the MS in the z-direction MF.

A z-direction MF was supplied to the MS. A bias of 2.5 V was applied to the bases of x/y-MSE. A bias of 5 V was provided to the collector of x/y-MSE, and a bias of 2 V was supplied to the additional collectors of x/y-MSE. At the same time, a bias of 1.5 was supplied to the bases of z-MSE, and a bias of 5 V was applied to the collectors of z-MSE. The digital multimeter detected the Vo of the x/y-MSE and z-MSE. Figure 20 displays three-axis Vo for the MS in the z-direction magnetic, where V_out_(x,z) is the x-axis Vo for the x/y-MSE in the z-direction MF; V_out_(y,z) is the y-axis Vo for the x/y-MSE in the z-direction MF and V_out_(z,z) is the z-MSE Vo in the z-direction MF. The V_out_(x,z) and V_out_(y,z) values in Figure 20 are very small. The slope of the curve V_out_(x,z) was 3.4 mV/T, and the slope of the curve V_out_(y,z) was 3.2 mV/T. In the z-direction MF, the MS had a cross-sensitivity of 3.4 mV/T (x-axis output) and a cross-sensitivity of 3.2 mV/T (y-axis output). The MS sensitivity in the z-direction MF was 119 mV/T, so the MS cross-sensitivity in z-direction MF was less than 2.9%.

Table 2 summarizes the performances of the magnetic sensor. The area of the x/y-MSE is 80 × 80 μm^2^, and the area of z-MSE is 120 × 120 μm^2^. The measurement range of magnetic field for the x-MSE, y-MSE and z-MSE is ±200 mT. The sensitivity of the x-MSE is 534 mV/T, and the sensitivity of the y-MSE is 525 mV/T. The sensitivity for the z-MSE is 119 mV/T. The cross-sensitivity of the x-MSE is less than 4.8%. The cross-sensitivity for the y-MSE is less than 4.7%, and the cross-sensitivity for the z-MSE is less than 2.9%. The output linearity for the x-MSE (coefficient of determination R^2^ = 0.9984), y-MSE (R^2^ = 0.9982) and z-MSE (R^2^ = 0.9995) is 99%. The power consumption of the x/y-MSE is 6 mW, and the power consumption of the z-MSE is 4 mW.

These micro magnetic sensors, proposed by Niekiel [9], Okada [11], Nejad [13], Bahreyni [14], were magnetic-resonant types that had a high sensitivity. The magnetic-resonant magnetic sensors required suspension structures and a high-actuated voltage to produce actuation and sensing, so the sensors had the disadvantages of a complicated fabrication, high-actuated voltage, high power consumption, and easy interference by environmental vibration. In this work, the micro magnetic sensor was without a suspension structure and was fabricated using the commercial CMOS process, so the sensor had the advantages of a low power consumption, easy fabrication and easy mass-production. Tseng [15] developed a one-axis micro-magnetic sensor using the CMOS process, and the sensor was a magnetic transistor type. The sensitivity of the magnetic sensor was 354 mV/T. Sileo [16] fabricated a Hall magnetic sensor, and its sensitivity was 0.03 V/T. Zhao [17] proposed a three-axis micro magnetic sensor manufactured using the MEMS technology. The magnetic sensor was composed of four magnetic transistors and a Hall element. The x-axis, y-axis and z-axis sensitivity for the magnetic sensor were 77.5 mV/T, 78.6 mV/T and 77.4 mV/T, respectively. In this work, the magnetic sensor was a magnetic transistor type, and the sensitivity of the sensor exceeded that of Tseng [15], Sileo [16] and Zhao [17]. 

## 5. Conclusions

The design and manufacturing of a three-axis MS base on the standard 180 nm CMOS technology were implemented. The MS was composed of an x/y-MSE and a z-MSE, where the z-MSE detected the MF in the z-direction and the x/y-MSE sensed the MF in the x- and y-direction. The x/y-MSE, which consisted of two magnetotransistors, designed four additional collectors that enhanced the mobility of carriers in the p-substrate and increased the MS sensitivity. The Sentaurus TCAD simulated the MS characteristic. The simulated results revealed that the MS sensitivity was 580 mV/T in the x-direction MF, 580 mV/T in the y-direction MF and 135 mV/T in the z-direction MF. The MS was an easy fabrication because it was without any post-CMOS process. The measured results depicted that the MS sensitivity was 534 mV/T in the x-direction MF, 525 mV/T in the y-direction MF and 119 mV/T in the z-direction MF. The measured results of the MS was in agreement with the measured results of the MS. Experiments showed that the MS cross-sensitivity in the x-direction was less than 4.8%, and its cross-sensitivity in the y-direction was less than 4.7%. The MS cross-sensitivity in the z-direction MF was less than 2.9%. Thereby, the MS had a low cross-sensitivity and an excellent sensitivity.

## Figures and Tables

**Table 1:** Summary of sensing principle and type for various micro magnetic sensors.

Authors	Sensing Principle	Type
Niekiel [9]	magnetic-resonance	1−axis
Chen [10]	giant magneto-impedance	1−axis
Okada [11]	magnetic-resonance	1−axis
Guo [12]	fluxgate	1−axis
Nejad [13]	magnetic-resonance	1−axis
Bahreyni [14]	magnetic-resonance	1−axis
Tseng [15]	magnetic-transistor	1−axis
Sileo [16]	Hall element	3−axis
Zhao [17]	magnetic-transistor + Hall element	3−axis
Yeh [18]	magnetic-piezoelectric	3−axis

**Table 2:** Summary of the performances for the magnetic sensor.

Characteristic	x/y-MSE		z-MSE
	x-MSE	y-MSE	
V_B_ voltage	2.5 V	2.5 V	1.5 V
V_C_ voltage	5 V	5 V	5 V
V_CA_ voltage	2 V	2 V	-
Area	80 × 80 μm^2^	combination with x-MSE	120 × 120 μm^2^
Measurement range	±200 mT	±200 mT	±200 mT
Sensitivity	534 mV/T	525 mV/T	119 mV/T
Cross-sensitivity	<4.8%	<4.7%	<2.9%
Output linearity	99%	99%	99%
Power consumption	6 mW	6 mW	4 mW

## Data Availability

Data sharing not applicable.
